# Evidence-Based Heatstroke Management in the Emergency Department

**DOI:** 10.5811/westjem.2020.11.49007

**Published:** 2021-02-26

**Authors:** Caitlin Rublee, Caleb Dresser, Catharina Giudice, Jay Lemery, Cecilia Sorensen

**Affiliations:** * University of Colorado School of Medicine, Department of Emergency Medicine, Aurora, Colorado; † Harvard Medical School, Beth Israel Deaconess Medical Center, Department of Emergency Medicine, Boston, Massachusetts; ‡ Los Angeles County and University of Southern California, Department of Emergency Medicine, Los Angeles, California

## Abstract

**Introduction:**

Climate change is causing an increase in the frequency and intensity of extreme heat events, which disproportionately impact the health of vulnerable populations. Heatstroke, the most serious heat-related illness, is a medical emergency that causes multiorgan failure and death without intervention. Rapid recognition and aggressive early treatment are essential to reduce morbidity and mortality. The objective of this study was to evaluate current standards of care for the emergent management of heatstroke and propose an evidence-based algorithm to expedite care.

**Methods:**

We systematically searched PubMed, Embase, and key journals, and reviewed bibliographies. Original research articles, including case studies, were selected if they specifically addressed the recognition and management of heatstroke in any prehospital, emergency department (ED), or intensive care unit population. Reviewers evaluated study quality and abstracted information regarding demographics, scenario, management, and outcome.

**Results:**

In total, 63 articles met full inclusion criteria after full-text review and were included for analysis. Three key themes identified during the qualitative review process included recognition, rapid cooling, and supportive care. Rapid recognition and expedited external or internal cooling methods coupled with multidisciplinary management were associated with improved outcomes. Delays in care are associated with adverse outcomes. We found no current scalable ED alert process to expedite early goal-directed therapies.

**Conclusion:**

Given the increased risk of exposure to heat waves and the time-sensitivity of the condition, EDs and healthcare systems should adopt processes for rapid recognition and management of heatstroke. This study proposes an evidence-based prehospital and ED heat alert pathway to improve early diagnosis and resource mobilization. We also provide an evidence-based treatment pathway to facilitate efficient patient cooling. It is hoped that this protocol will improve care and help healthcare systems adapt to changing environmental conditions.

## INTRODUCTION

Climate change is causing a global increase in average temperatures as well as an increase in the frequency, duration, and intensity of extreme heat events,^1^–^3^ resulting in unprecedented levels of exposure to heat. Between 2000 and 2016, an estimated 125 million additional adult Americans were exposed to heat waves, and in the year 2017 alone, the majority of Americans experienced temperatures that were the hottest recorded.^4^,^5^ The number of days with dangerously elevated temperatures are projected to increase in coming years.^6^ This will create significant challenges for exposed populations, healthcare systems, and public health officials leading community prevention and response efforts.^7^ Emergency departments (ED) are likely to treat increasing numbers of patients affected by extreme heat.^8^ According to the Centers for Disease Control and Prevention, heat-related illnesses are the leading cause of weather-related death in the United States.^9^

Heatstroke, the most serious heat-related illness, is a medical emergency that requires rapid recognition and treatment to prevent permanent morbidity and mortality.^10^ The hallmark of heatstroke is the combination of central nervous system dysfunction and elevated core body temperature, defined as over 40 degrees Celsius.^11^ The presenting symptoms of heatstroke can mimic many other illnesses including sepsis, ischemic stroke, and toxicologic emergencies, particularly if a core body temperature is not obtained. On average, 618 deaths are reported per year in the US due to environmental heat^12^; however, this is likely a gross underestimate of the true extent of heat-related illness as comorbid diseases that are exacerbated by heat exposure are often erroneously reported as the primary diagnosis, thus concealing the role of heat as an inciting factor.^13^

Exposure to elevated ambient temperatures coupled with increased metabolic activity may result in heat illness if the individual has exhausted physiological compensatory mechanisms and is unable to take behavioral steps to cool down.^14^ Heat stress initially leads to activation of compensatory mechanisms such as sweating, which help maintain stable core temperature, but eventually lead to consumption of fluid and electrolyte reserves. Once these internal and behavioral mechanisms are overwhelmed, core temperature can rise precipitously. Left unchecked, elevated core temperature can result in catastrophic multisystem illness characterized by renal injury, liver injury, vascular inflammation, coagulopathy, airway spasms, disruption of homeostatic thermoregulation, and central nervous system dysfunction and death.^15^

Any person exposed to high environmental temperatures is at risk of heatstroke, but specific populations are at a comparatively higher risk of experiencing adverse health outcomes. Heat poses serious risks for children, older adults, pregnant women, and those with chronic health conditions such as cardiovascular, respiratory, renal, or psychiatric disease. Heat illness is also a disease of socioeconomic vulnerability and occupational vulnerability.^8^,^16^ Communities in rural areas as well as dense urban settlements (heat islands) are at higher risk as well as certain demographics of workers, which include outdoor workers employed in agriculture and construction, first responders, military personnel, and others.^5^

Medications that interfere with salt and water balance and circulatory function place individuals at even higher risk. These medications include but are not limited to diuretics, anticholinergic agents, and beta-blockers, as well as medications that interfere with centers of thermoregulation such as selective serotonin reuptake inhibitors and anti-dopaminergic medications.^17^ Heatstroke can result from either environmental exposure (classic heatstroke) or intense physical activity (exertional heatstroke), but in both cases early identification and rapid intervention are critical to survival and neurologic outcome. Older adults are at risk for classic heatstroke while younger individuals tend to be affected by exertional heatstroke.

**Population Health Research Capsule**

What do we already know about this issue?

*Heatstroke is a life-threatening, time-sensitive condition that requires significant resources to treat*.

What was the research question?

What are the processes and current standards of care in the literature for the acute management of heatstroke?

What was the major finding of the study?

*Recognition, rapid cooling, and supportive care were key steps to treatment, yet no scalable process was found*.

How does this improve population health?

*Our proposed heatstroke treatment pathway may assist healthcare systems to adapt to a changing climate and protect populations at increased risk*.

Emergency departments have alert systems for other high-risk, time-sensitive conditions to appropriately allocate human and hospital resources in a timely manner to improve patient outcomes. For example, sepsis and cardiac alerts are a component of quality incentive metrics for many EDs and have been shown to improve mortality.^18^–^20^ Stroke alerts expedite imaging and timely intervention.^21^,^22^ With these time-sensitive conditions, emergency medical services (EMS) also participates in identifying and initiating treatment by alerting treatment teams prior to hospital arrival to prepare for rapid, aggressive treatment. The time sensitivity of heatstroke and the risk of severe complications warrants a similar approach. Currently, national EMS protocols exist for hyperthermia,^23^ but there are no mandates for implementation and each state determines its own protocols (if any). Similarly, many EDs lack protocols and have variable guidelines for management of heat-related illness, which vary by institution.^24^

The current lack of standardization and deployment of evidence-based protocols presents an opportunity to save lives and improve patient outcomes by instituting system-based approaches to healthcare delivery for heatstroke patients. Here, we review current standards of care for the emergent management of heatstroke and propose an evidence-based algorithm to expedite care and improve recognition and treatment of this condition.

## METHODS

### Study Eligibility

The primary topical focus for all articles included in the study was heatstroke, defined as the combination of elevated core body temperature and altered mental status due to either ambient temperature, exertion, or both. Secondary topical foci included acute management of heatstroke and/or early outcomes associated with management. Studies were excluded for the following reasons: 1) non-English language; 2) non-human subjects; 3) full text not available; 4) qualitative studies unless high-level consesus panel recommendations; and 5) topical focus only on prevention or physiology descriptions, rather than acute treatment.

We included all quantitative studies. In addition, we included qualitative studies published as a consensus recommendation from a major health body such as the World Health Organization, US Occupational Safety and Health Administration, EMS, or military. Quantitative articles included published articles and articles in press, conference papers, editorials, reviews, case reports, and case series. Included articles were written in English and had full text available.

### Study Identification

We performed searches for scientific articles addressing the acute management of heatstroke using PubMed and Embase databases without date restrictions. This review did not meet eligibility criteria for a PRISMA systematic review or meta-analysis as it included two databases.^25^ Search terms are available in the Supplemental Methods. All English-language articles meeting the heatstroke topical and situational criteria were included. The search strategy was designed in collaboration with a health sciences medical librarian with the goal of identifying articles that addressed the rapid recognition and treatment of heatstroke in prehospital, ED, or critical care settings. Additional searches of the non-peer-reviewed medical literature were performed to capture prehospital protocols and expert opinion. Additional articles were added after independent review of the references of articles identified during the literature search.

### Study Selection

After articles were identified via the initial search strategy, duplicates were removed, and titles and abstracts were screened for relevance and consistency with the inclusion criteria. Each article was read and assessed by two independent, blinded physician reviewers (CR, CD, CG, CS). Any discrepancies were resolved by a third blinded physician-author reviewer. We tracked inter-rater reliability for inclusion and exclusion criteria and the Grading of Recommendations Assessment, Development, and Evaluation (GRADE) criteria. We used EndNote bibliographic manager to assist in the review. Additional articles were found from bibliographic review of selected studies for inclusion.

### Data Extraction

Reviewers extracted information on author, year, study design, study setting (prehospital, ED, intensive care unit [ICU]), population, topical focus (recognition, cooling, management, systems), classic vs exertional heatstroke, cooling method, prognosticators if measured, and outcome. Notable case complications were also recorded. Quantitative studies were assessed by reviewers using the GRADE criteria, which included very low, low, moderate, and high quality.^26^

### Data Synthesis

We performed a descriptive thematic analysis due to the heterogeneous nature of the articles and developed a management pathway based on evidence in the literature.

## RESULTS

### Overview

**Figure 1:**
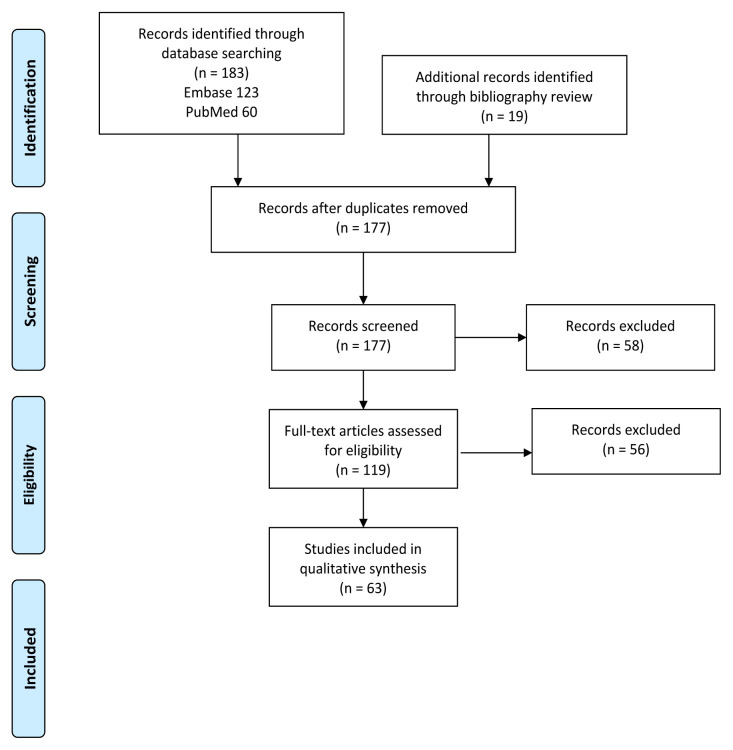
Flow chart for selected studies on heatstroke management.

Of the 183 articles identified in the search, 25 duplicates were removed and 58 were excluded by title and abstract review (Figure 1). In total, 63 articles met full inclusion criteria after full-text review and were included for analysis (Supplemental Results Table S1). Studies were primarily excluded due to lack of topical focus on heatstroke or acute management. There was discrepancy between reviewers for 14 studies from the database that required a third reviewer. Of those studies, nine were excluded and five were included. Studies included all years with relevant results, with publication dates ranging from 1956 to 2020.

Quality of studies varied. Of the included studies, 25 were assessed to be very low quality, 25 were low quality, and 13 were moderate quality. No studies were ranked as high quality. There was discrepancy in 17 of 63 studies (27%) between reviewers. All were one level of evidence off and resolved by a third blinded reviewer. The majority of moderate-quality studies were found through the search of references (10 of 13). Just over half of the included studies (34) were case reports or case series. Two case reports also included a review of current standards of care.^27^,^28^ In total, 16 reviews, primarily unstructured, were identified, and 15 other studies ranged from opinion pieces to observational studies.

Exertional heatstroke was addressed in 38 studies, classic heatstroke in 12 studies, and both types in 13 studies. Patients in specific case reports and series were more frequently male than female.. More than half of studies (39 of 63) focused on a single care setting rather than across all three settings—prehospital, ED, and ICUs; management included prehospital care in 37 studies, EDs in 33 studies, and ICUs in 25 studies.

Numerous patient populations from infants^29^ to older adults^30^–^32^ were identified in this review of heatstroke. Pediatric athletes^27^,^33^–^35^ and pediatric vehicular heatstroke cases were discussed.^36^,^37^ Occupational heatstroke was described in construction workers,^38^,^39^ a baker,^40^ gold miners,^41^ and an aluminum smelter pot room process-control operator.^42^ Environmental circumstances including heat waves^29^,^31^,^40^,^43^,^44^ and sunbathing^45^ were identified as risks, as was non-endemic heat due to a dry sauna exposure.^46^ Exertional heat stress was described in runners,^47^–^50^ individuals along the US-Mexico border,^28^ participants in the Hajj pilgrimage to Mecca,^51^ and military personnel.^52^–^61^ While many predisposing factors have previously been identified,^62^ specific cases highlighted hypohydrosis disorder,^63^ antipsychotic medications,^31^ and social determinants of health such as poor housing^29^ and lack of indoor cooling^43^ as contributory to heatstroke.

Populations represented were from geographically diverse settings. Countries/regions included Pakistan,^64^ China,^65^ India,^55^,^66^ Saudi Arabia,^51^,^63^ Australia,^50^ Puerto Rico,^53^ the United Kingdom,^48^ Japan,^34^ Israel,^67^ Nigeria^36^ and other African countries,^41^ France,^31^ and the United States of America.^32^,^43^,^68^,^69^

### Key Steps in Heatstroke Management

We identified three principle themes for clinical management: recognition, rapid cooling, and supportive care (Table).

#### Recognition

Heatstroke recognition was highlighted in 23 studies. Topical focus was cooling in almost all studies (55 of 63). Cooling methods included removal from the hot environment, fluid resuscitation, cold water or ice water immersion, application of cold packs, evaporative cooling with water and fans, internal cooling (gastric, bladder, and/or rectal), endovascular cooling,^28^,^40^,^54^,^70^ and extracorporeal membrane oxygenation (ECMO)-based cooling.^71^

#### Rapid Cooling

Preferred cooling treatments varied, with no clear prevailing recommendation. A previous systematic review (2007) showed no definitive data to guide specific cooling approaches.^24^ In one study in which cooling rates were compared,^72^ cold water immersion was considered the gold standard; this is consistent with the Wilderness Medicine Society Grade 1A recommendations for cold water immersion of heatstroke patients.^73^,^74^ Other studies by the same author supported cold water immersion.^75^,^76^ Earlier work found no statistically significant difference between ice water immersion and cold water immersion.^77^ One study recommended ice water immersion.^78^ Other than rate of cooling, a primary consideration was mental status and other monitoring required in these patients. In intubated, obtunded patients, a cooling catheter was placed^70^ or evaporative cooling was preferred for patient safety.^49^,^60^

#### Supportive Care

Laboratory management was discussed in 23 studies with variation in values reported. One study found sodium >145 was an independent risk factor for death^44^; in contrast, low sodium was reported to be common in other cases.^42^,^65^ A case series found both high and low values of potassium, sodium, calcium, and phosphorus.^65^ Aspartate aminotransferase >1000 units was associated with death.^41^ Elevated troponin T and creatine kinase were also noted in patients.^49^ Lactate, troponin I, and creatinine were significantly elevated in non-survivors of classic heatstroke as compared with survivors, although time to cooling in the non-survivor group was significantly longer.^31^ Resuscitation guidance was the focus of two studies,^51^,^65^ which recommended Foley catheters to monitor urine output and avoidance of over-resuscitation due to risk of pulmonary edema.^51^ Only four studies addressed systems-based approaches to heatstroke; all focused on exertional heatstroke.^50^,^57^,^60^,^79^

Many of the reported patient outcomes consisted of complete recovery followed by discharge from the hospital.^30^,^36^,^38^,^48^,^49^,^53^–^57^,^60^,^68^,^70^,^80^–^82^ Several patients required a prolonged hospitalization up to 75 days.^39^,^63^,^83^ Notable case sequelae were ventricular tachycardia,^39^ aspiration,^40^,^60^ cerebral edema,^53^ seizures,^38^,^55^ residual neurological deficits,^42^,^59^,^63^,^66^ acute liver failure that required transplantation^46^,^83^,^84^ or supportive care,^45^ and death.^27^,^29^,^31^,^34^,^43^,^47^,^59^,^65^,^66^,^68^ It was noted that many patients arrived via EMS, yet cooling was often delayed.^31^,^32^,^46^,^47^,^58^,^67^ However, none of the articles or guidelines that were reviewed described a scalable system-based EMS and ED process, alert, pathway, or algorithm to expedite early identification and intervention.

## DISCUSSION

This literature review examined current available English-language literature on the recognition and management of acute heatstroke. This review did not identify any standardized, systematic approach for EMS or ED treatment of heatstroke. These results are consistent with findings of a previous review.^24^ Available guidelines tend to emphasize “rapid cooling” without exploring the specific operational steps that are necessary to ensure this occurs efficiently and consistently in practice, despite the fact that most deaths are attributable to delays in prehospital or ED care. There is some evidence that identification of patients with heatstroke may be a limiting factor; consideration of elevated indoor and outdoor temperatures, membership in vulnerable groups, and recent or ongoing increased metabolic demand^37^ were shown to improve detection, but currently are not applied in a systematic fashion.

The review supports the efficacy of standard emergency medicine (EM) management of heatstroke. However, it appears that the application of these techniques is variable, as is timely identification of at-risk patients. Here, we propose an ED heatstroke pathway to facilitate rapid identification and timely intervention for these critically ill patients.

### Treatment Approach

Rapid identification and initiation of treatment in patients with heatstroke is a core component of EM training yet remains difficult to implement in many settings. Heatstroke is an uncommon diagnosis that is time and resource intensive; early diagnosis is both challenging and essential. Published literature demonstrates reduced morbidity and mortality with prompt action and provides evidence for key clinical actions in heatstroke management.

#### Early Recognition and Core Temperature

Early recognition is consistently emphasized in the published literature. Military events and athletic events frequently have protocols in place during warm weather days. Other variables of heat stress beyond temperature were inconsistently incorporated, such as wet-bulb globe temperature.^69^ Heat index (temperature and humidity) was used in the algorithm similar to previous work.^85^ Time of the year and active heat advisories were also considered to address exposure risks.

Elevated core body temperature is a crucial cue to responders to initiate cooling. Thus, early rectal temperature measurement^35^,^54^,^68^,^86^ was emphasized in the algorithm; empiric treatment is also an option if high suspicion exists and it is not possible to obtain a core temperature.^77^ Failure to recognize heatstroke was life-threatening.^46^,^58^,^67^ In contrast, patients who were rapidly cooled frequently had rapid reversal of mental status changes^30^,^49^ and in some cases were discharged from the ED. In one study of 274 cases of exertional heatstroke, there was 100% survival with on-site immersion in cold water.^81^

#### Treat or Transport? Advantages of Cooling Prior to Transport or Transfer

Cooling was recommended prior to transfer in several publications, with recommendations to continue cooling during transport if possible.^35^,^49^,^50^,^79^ Time was the main driver of this recommendation, with a goal of less than 30 minutes^69^ or less than 60 minutes to cooling.^86^ Delays in cooling contributed to adverse outcomes.^47^,^31^,^32^,^67^

On-site cooling is important in rural settings; the literature favors continued attempts at cooling prior to transport until temperature is controlled or until all means of cooling are exhausted. Urban populations were more represented in the literature^32^,^43^,^68^ than rural populations, but some of the methods of cooling described in the literature may be most applicable in remote settings. For example, sites with air transport can use the downdraft of a helicopter as a fan to evaporate cool water.^82^

#### Allocation of Human and Medical Resources

Unique and specialized resources such as cooling devices as well as multiple specialties and staff resources are required to manage heatstroke. A target goal of 39°C was chosen as other studies demonstrate safety between 38.3–39°C.^24^,^74^ Early initiation of the heat response algorithm is expected to facilitate appropriate care early in the treatment timeline. We believe a protocol encourages discussion of resources available at individual EDs before the first patient arrives, as many methods are available for effective cooling: body bag,^30^ tarp,^80^ helicopter downdraft,^82^ endovascular,^70^ and ECMO.^71^,^87^ Other more robust responses such as a dedicated heatstroke unit^55^ or an on-site, field-deployed body cooling unit^49^ may apply in certain settings.

A proportion of patients required intubation and definitive airway management. In one study, non-survivors were more likely to have been intubated in the ED than survivors.^32^ When mentioned, rocuronium^39^ and succinylcholine^27^ were both used in patients with no discussion on preference of one over the other. While there is a theoretical basis to support the use of a nondepolarizing agent such as rocuronium, which avoids possible heat generation during fasciculations and results in longer duration neuromuscular blockade that may reduce metabolic heat generation, no clear evidence supports the recommendation of rocuronium in favor of succinylcholine.

Transfer to a liver transplant center was included in the algorithm as a consideration due to several cases of acute liver failure secondary to heatstroke.^46^,^83^,^84^ There was one case of a teenager who died secondary to acute liver failure and disseminated intravascular coagulation (DIC) with no transplant.^27^ Of the three cases identified in the review in which patients underwent transplant, two athletes with exertional heatstroke required transplant on day three of admission^83^,^84^ and one required transfer to a transplant center on day three followed by transplant on day six.^46^

#### At-risk Populations

Certain populations appear to be at elevated risk. The populations identified in this review include older adults, who tended to present with classic heatstroke, and athletes and military personnel, who tended to present with exertional heatstroke. Identification of at-risk populations can help educators inform the general public, public health agencies, occupational health agencies, and first responders with regard to identification of potential heatstroke patients.

Classic heatstroke was predominantly described in urban environments; patients tended to be older and suffer worse outcomes^32^ than those described for exertional heatstroke.^81^ The 1959 heat wave in Melbourne,^29^ the 1995 heat wave in Chicago,^43^ and the 2003 heat wave in France,^31^,^40^,^44^ exposed large numbers of urban dwellers to sustained high temperatures with tragic consequences. Hypernatremia was an independent risk factor for death in the heat wave in France, which was associated with advanced age.^44^ Patients with classic heatstroke tended to have underlying comorbidities that placed them at elevated risk, although mortality rates varied widely from 17%^68^ to 63.6%.^31^

Most descriptions of exertional heatstroke involved athletes and military personnel. Among runners, intermediate-skill runners were more often described as suffering from heatstroke as compared to novice or elite runners.^48^,^50^ Hyperkalemia (9 milliequivalents per liter), elevated creatinine, rhabdomyolysis, acidosis, and elecrocardiogram changes were associated with one runner fatality.^69^ Two publications described non-athletic, nonmilitary cases of exertional heatstroke. One case report highlighted a male along the US border who had been walking for 24 hours.^55^ A second publication described a male construction worker who had persistent ventricular tachycardia until he was cooled.^39^

Men were more represented than women in the literature. Larger body mass may contribute, although males do not appear to be at higher physiologic risk once cooling is initiated. A study of exertional heatstroke in runners found no statistical difference in cooling rate based on initial temperature, age, or gender.^81^ The discrepancy in publication volume may be a function of historical gender patterns in outdoor work and military activities; it should be noted that women are also at risk in similar situations, as described in a case report of an 18-year-old female military recruit who suffered rhabdomyolysis and a two-week hospitalization before being discharged neurologically intact.^61^

#### A Community-based Approach to Heatstroke

Heatstroke is a preventable yet under-recognized medical emergency. Only four studies clearly addressed system-based changes^50^,^57^,^60^,^79^ despite previous calls for systemic approaches to address heat-related illness, particularly in EM.^88^ None of these studies addressed classic heatstroke or the impact of heat waves, despite the fact that these patients are often more vulnerable, present later, and suffer worse outcomes, and the fact that heat wave frequency and intensity is projected to increase as a result of climate change. This situation represents a significant opportunity for communities to reduce health harms and direct and indirect healthcare costs associated with extreme heat from lost productivity, worker absenteeism, medications, and healthcare utilization.^89^

While the present study did not address prevention, the results are useful for stakeholders working to expand syndromic surveillance and warning systems. Real-time surveillance has already demonstrated success for monitoring deaths and public health interventions during a heat wave.^90^ Heat early warning systems have reduced heat exposure risks in communities by evaluating healthcare data and heat index values for heat alert processes.^85^ Multidisciplinary teams with representatives from athletics, public health, climate sciences, emergency management, energy, city planning, and meteorology have had meetings using the National Integrated Heat Health Information System to better prepare and adapt to heat.^91^ Furthermore, these efforts demonstrate an active commitment to addressing climate change and to improving social and environmental determinants of health,^29^,^43^ and resultant health inequities. Thus, final recommendations focus on an integrated approach both in the ED and in the community to facilitate heat-related illness education, recognition, and treatment.

### Heat Alert Algorithm

**Figure 2:**
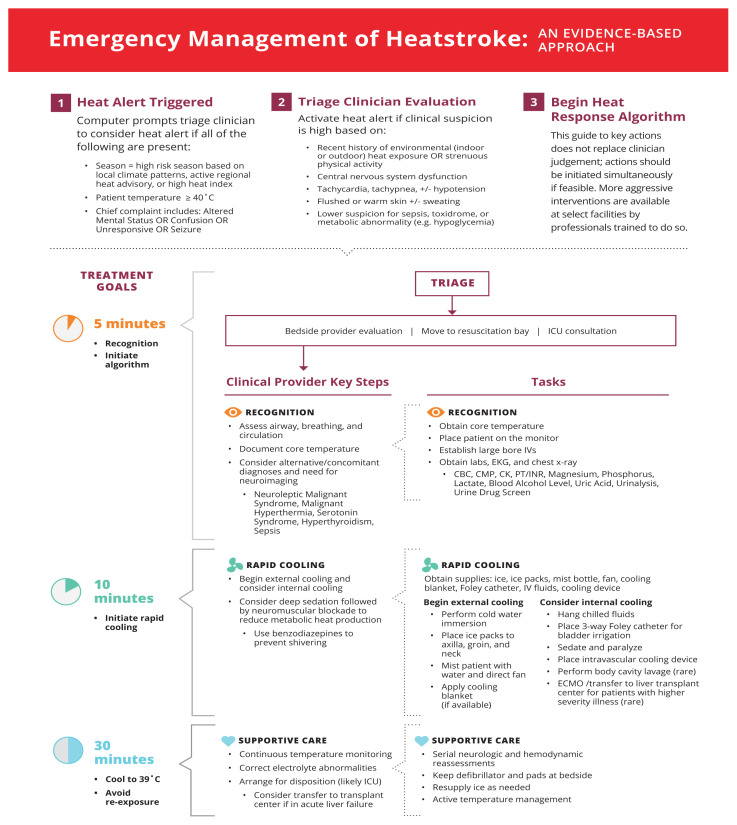
Management of heatstroke in the emergency department. *CBC*, complete blood count; *CMP*, comprehensive metabolic panel; *CK*, creatine kinase; *PT/INR*, prothrombin time/international normalized ratio; *ECMO*, extracorporeal membrane oxygenation; *ICU*, intensive care unit.

We developed an alert process and treatment algorithm to facilitate critical care delivery in EDs (Figure 2). The algorithm is based on available evidence from the published literature regarding presentation, critical interventions, and time-dependence of interventions. Seasonal timing is appropriate for use in the Northern hemisphere temperate zone for peak heat illness.^92^ Details should be adjusted to match local conditions.

In Step 1 of the algorithm, a heat alert flag integrates information on current environmental conditions, body temperature, and patient complaints and prompts triage staff to consider whether the patient has heatstroke. In Step 2, the heat alert expedites clinical evaluation by a trained health professional to assess for other underlying etiologies and ultimately triggers or ceases the continuation of the algorithm. Step 3 is the heat response guide for members of the healthcare team to perform within 30 minutes of evaluation.

### Recommendations

- Health systems need to implement heat alert systems and train relevant staff members
- Include prehospital providers and EMS networks in early identification, early communication, and treatment of heat illness
- Increase public health messaging around risks of endemic and acquired heat illness especially among vulnerable populations
- Increase syndromic surveillance and improve heat warning systems

## STRENGTHS AND LIMITATIONS

This study has a few strengths. A rigorous search strategy was developed in partnership with a research librarian across two large databases. Next, the literature included in the review spanned 64 years with results that represented populations from infants to older adults and incorporated a spectrum of occupational as well as endemic and acquired heatstroke cases across a wide range of geographic areas. The mix of study settings also incorporated the expertise of prehospital, emergency care and critical care providers, which strengthened the management approach.

Several limitations remain. The majority of studies were of low-quality evidence as case reports or case series. This may limit validity and allows for confounding factors for management suggestions. For example, time to cooling rather than specific laboratory prognosticators may influence patient outcomes the most. While many populations were represented, missing vulnerable populations, such as prisoners, were not included. These populations may benefit from systemic changes and protocols the most and will be important to include in further implementation of scientific research on efficacy and outcomes of protocols.

## CONCLUSION

Rapid recognition and management of heatstroke is critical for the healthcare system to successfully adapt to the increases in frequency, intensity and duration of heat waves as a result of climate change. The proposed heat alert algorithm is intended to help ED and prehospital teams identify heatstroke patients, implement critical treatments, and allocate resources in a timely fashion. The process presented here is a template for evidence-based clinical practice and may help institutions meet the standard of care for patients with life-threatening heat-related illnesses. Improved recognition and treatment of heatstroke has the potential to reduce mortality and neurological complications and support vulnerable patients in a rapidly warming world.

## Supplementary Information





## Figures and Tables

**Table:** Principle themes identified in literature review concerning heatstroke.

Topic	Recommended action
Recognition	Maintain high clinical suspicion Weather awareness Measure core body temperature
Rapid cooling	Initiate immediate external cooling Early decision regarding invasive cooling
Supportive care	Emphasis on airway, breathing, circulation Monitor for and correct metabolic derangements
