# Supplementary material for: Evidence-Based Heatstroke Management in the Emergency Department
Source: West J Emerg Med. 2021 Feb 26;22(2):186–95. doi: 10.5811/westjem.2020.11.49007 (PMC7972371; doi:10.5811/westjem.2020.11.49007)
Supplement: Supplementary file 1 [file wjem-22-186-s001.docx]

**Supplemental Methods**

Search terms used for the review

**PubMed**

N=60

("Heat Stroke"[Mesh] OR "heat stroke"[tw] OR "heatstroke"[tw]) AND ("Emergency Treatment"[Mesh] OR "acute care"[tw] OR "acute medical care"[tw] OR "emergency care"[tw] OR "emergency health care"[tw] OR "emergency medical care"[tw] OR "patient care, prehospital"[tw] OR "prehospital care"[tw] OR "Evidence-Based Emergency Medicine"[Mesh] OR "evidence based emergency medicine"[tw] OR "evidence-based emergency medicine"[tw] OR "advanced cardiac life support"[tw] OR "bystander cpr"[tw] OR "cardio pulmonary resuscitation"[tw] OR "cardiopulmonary resuscitation"[tw] OR "chest compression"[tw] OR "resuscitation"[tw] OR "resuscitation orders"[tw])

**EMBASE**

N= 123 results

('heat stroke'/exp OR 'heat stroke':ti,ab OR 'heatstroke':ti,ab) AND ('emergency care'/exp OR 'acute care':ti,ab OR 'acute medical care':ti,ab OR 'emergency care':ti,ab OR 'emergency health care':ti,ab OR 'emergency medical care':ti,ab OR 'patient care, prehospital':ti,ab OR 'prehospital care':ti,ab OR 'evidence based emergency medicine'/exp OR 'evidence based emergency medicine':ti,ab OR 'evidence-based emergency medicine':ti,ab OR 'resuscitation'/exp OR 'advanced cardiac life support':ti,ab OR 'bystander cpr':ti,ab OR 'cardio pulmonary resuscitation':ti,ab OR 'cardiopulmonary resuscitation':ti,ab OR 'chest compression':ti,ab OR 'resuscitation':ti,ab OR 'resuscitation orders':ti,ab)

**Supplemental Results**

See Table S1 Summary of Reviewed Articles.
